# Targeted Therapy for Gut Microbiota: Candidates for a Novel Strategy to Ameliorate Type 2 Diabetes Mellitus

**DOI:** 10.1111/1751-7915.70283

**Published:** 2025-12-15

**Authors:** Jiangyan Wang, Yaofei Wei, Dongmian Chen, Xia Li, Hao Zhang, Shuo Feng, Shenghua Lu, Juan Yang, Qi Zeng, Xingxiang He, Lei Wu

**Affiliations:** ^1^ Department of Gastroenterology, Research Center for Engineering Techniques of Microbiota‐Targeted Therapies of Guangdong Province The First Affiliated Hospital of Guangdong Pharmaceutical University Guangzhou China; ^2^ Guangdong Provincial Key Laboratory for Research and Evaluation of Pharmaceutical Preparations Guangzhou China; ^3^ School of Foreign Languages of Guangdong Pharmaceutical University Guangzhou China

**Keywords:** faecal microbiota transplantation, gut microbiota, prebiotics, probiotics, synbiotics, type 2 diabetes mellitus

## Abstract

Type 2 diabetes mellitus (T2DM) poses a significant threat to public health and is associated with the gut microbiota. Gut microbiota modulators, including probiotics, prebiotics, and synbiotics, together with faecal microbiota transplantation (FMT), can restore the gut microbiota in patients and are recognised as powerful modulators of this ecosystem. Consequently, gut microbiota modulators are promising in the prevention and treatment of T2DM. The roles and mechanisms by which these therapeutic approaches target the gut microbiota in patients with T2DM warrant further investigation and elucidation. Key potential mechanisms associated with gut microbiota regulation include the modulation of gut microbiota composition alteration of gut microbiota metabolites, enhancement of intestinal barrier function, and suppression of inflammation. This study provides a comprehensive review of the relationship between the gut microbiota and T2DM, presents promising research findings and controversial issues, emphasises the potential roles and mechanisms of the gut microbiota in T2DM, and investigates the factors influencing the therapeutic efficacy of FMT. This review serves as a valuable reference for future studies on FMT.

## Graphical abstract

Type 2 diabetes mellitus (T2DM) poses a significant threat to public health and is linked to the gut microbiota. Probiotics, prebiotics, and synbiotics, together with fecal microbiota transplantation (FMT), can restore the gut microbiota in type 2 diabetes mellitus (T2DM) patients.

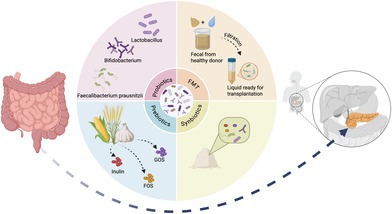

## 1 Introduction

Type 2 diabetes mellitus (T2DM) is a common metabolic disorder characterised by a sustained increase in global prevalence over the past five decades, with a prominent shift in epidemiological distribution from Western countries to the Western Pacific Region, encompassing parts of Asia and Africa (Roden and Shulman 2019). According to data from the International Diabetes Federation, approximately 537 million people worldwide were living with diabetes in 2021, accounting for 10.5% of the global population, with associated healthcare expenditure reaching USD 966 billion. It is projected that by 2030, the number of affected individuals will increase to 643 million (11.3% of the global population), and by 2045, this figure is expected to increase further to 783 million (12.2% of the global population), with healthcare costs exceeding USD 1054 billion (Hossain et al. 2024). T2DM accounts for approximately 90% of all diabetes cases. Currently, there is no definitive cure for diabetes, and glycemic control and symptom alleviation depend predominantly on conventional pharmacological interventions. Poor long‐term glycemic control may lead to severe complications and pose significant risks to patient safety (Yan et al. 2022). Moreover, the prolonged use of diabetes medications is often associated with considerable side effects. Therefore, the development of therapeutic strategies with fewer adverse effects and improved curative efficacy is of great importance.

The gut microbiota is characterised by substantial diversity and exerts profound regulatory effects on human physiology (Vemuri et al. 2020). A delicate balance is maintained between beneficial and harmful bacteria within the gut microbiota; when this equilibrium is disrupted, resulting in an increase in harmful bacteria and a decrease in beneficial bacteria, disease may ensue. The gut microbiota has a broad potential for application in the prevention, diagnosis, and treatment of various diseases. Studies have indicated that dysbiosis of gut microbiota is associated with T2DM (Chen, Liu, et al. 2023). Substantial research has demonstrated significant alterations in the gut microbiota of T2DM patients compared with healthy individuals (Ting Ye et al. 2020; Wu et al. 2010; Wu and Park 2022). Commensal fungi and opportunistic pathogens stimulate the local immune system and increase intestinal permeability, leading to a “leaky gut”. This, in turn, triggers systemic inflammation and contributes to the development of insulin resistance (IR) (Chen, Chen, and Fu 2023). Furthermore, metabolites produced by the gut microbiota, including short‐chain fatty acids (SCFAs), bile acids (BAs), and branched‐chain amino acids (BCAAs), have been implicated in the pathogenesis of T2DM (Wu, Yang, et al. 2023). Consequently, modulating the metabolites of the gut microbiota is regarded as an effective approach for treating T2DM.

Gut microbiota modulators include probiotics, prebiotics, synbiotics, and faecal microbiota transplantation (FMT). Previous studies indicated that gut microbiota modulators can effectively mitigate obesity, T2DM, and other metabolic diseases (Li et al. 2021). Probiotics include a variety of beneficial microorganisms, whereas prebiotics are organic compounds that selectively enhance the metabolism and proliferation of beneficial bacteria within the body, thereby promoting host health. Synbiotics are combinations of prebiotics and probiotics (da Silva et al. 2021). FMT is an innovative therapeutic strategy that uses the healthy gut microbiota to enhance the microbiota of patients. Washed microbiota transplantation (WMT), an adaptation of FMT, involves repeated washing of the microbiota to eliminate harmful substances, thereby improving therapeutic efficacy and minimising adverse effects (Zhang, Lu, et al. 2020). FMT has demonstrated significant efficacy in treating various conditions, including *Clostridioides difficile* infection (Lessa et al. 2012), obesity (Allegretti et al. 2020), and metabolic syndrome (Kootte et al. 2017). The therapeutic potential of FMT for diabetes has been discussed in several studies. In mouse models of T2DM, FMT restored the structure of the gut microbiota, reduced inflammatory responses, mitigated pancreatic islet damage, and improved IR, leading to decreased glycolipid levels (Zhang, Li, et al. 2020; Wang and Bai 2020). Correspondingly, clinical studies have shown that FMT markedly enhances IR, reduces body mass index (BMI), and alters the composition of the gut microbiota in patients with T2DM through colonisation by donor‐derived microbiota (Wu, Zhang, et al. 2023). This review aims to elucidate the relationship between the gut microbiota and T2DM while summarising the mechanisms of action and advances in research related to FMT in this context.

## 2 Changes in the Composition of the Gut Microbiota With T2DM

### 2.1 Differences in the Gut Microbiota Between Patients With T2DM and Healthy Individiuals

A substantial number of studies have confirmed altered gut microbiota in patients with T2DM compared with that of healthy individuals (Table 1). A decline in the core beneficial microbiota represented the predominant trend. *Bifidobacterium* abundance was significantly reduced in studies conducted in China and Iran (Wu et al. 2010; Sedighi et al. 2017). As a gut probiotic, its reduction may be associated with intestinal barrier impairment in patients with T2DM (de Cossío et al. 2017). *Faecalibacterium prausnitzii* was decreased in Iranian studies as well as in Asian studies (Wu and Park 2022; Navab Moghadam et al. 2017). *F. prausnitzii* is a major producer of SCFAs, and its reduction may affect insulin sensitivity (Ganesan et al. 2018). The phylum Firmicutes was reduced in Danish and Chinese T2DM patients with obesity (Ting Ye et al. 2020; Larsen et al. 2010), suggesting an association between the phylum Firmicutes and metabolic disorders. Furthermore, a large‐sample study from the United States revealed a reduction in microbial α‐diversity (e.g., Chao1 index) (Park et al. 2023), reflecting a general reduction in gut microbiota diversity in T2DM patients. Significant differences in the composition of gut microbiota exist across different regions and ethnic groups, which are primarily attributed to differences in dietary patterns and regional lifestyle factors (e.g., living habits). Notably, a mathematical model on the basis of metagenomic profiles demonstrated a high degree of accuracy in identifying T2DM in Europeans, although it was less effective in the Chinese population, indicating that the metagenomic markers for T2DM may differ between these groups (Karlsson et al. 2013). Wang et al. (2017) identified significant differences in the gut microbiota between patients with T2DM and healthy individuals and among various ethnic groups in Northwest China. Similarly, one of the largest studies to date explored the gut microbiomes of T2DM, prediabetic, and healthy populations across diverse geographic and ethnic backgrounds by integrating 8117 gut microbial macrogenomic samples from participants in multiple countries, including the United States, Israel, Switzerland, Finland, Denmark, Germany, France, and China. This study found substantial differences in gut microbiomes across geographic, racial, and ethnic groups (Mei et al. 2024).

**TABLE 1:** Differences in the gut microbiota between patients with type 2 diabetes mellitus and normal individuals.

Country	Research object	Research method	Compared with the control group, changes in the gut microbiota in patients with T2DM	References
China	Patients with T2DM (*n* = 16)	RT PCR	*Bacteroides vulgatus* and *Bifidobacterium*↓ *Clostridium leptum* subgroup↑	Wu et al. (2010)
Iran	Patients with T2DM (*n* = 18)	RT qPCR	*Bifidobacterium*↓ *Lactobacillus*↑	Sedighi et al. (2017)
Iran	Patients with T2DM (*n* = 18)	RT qPCR	*Faecalibacterium prausnitzii* ↓	Navab Moghadam et al. (2017)
Denmark	Patients with T2DM (*n* = 18)	RT qPCR	Phylum Firmicutes and class Clostridia↓ Class Betaproteobacteria↑	Larsen et al. (2010)
Japanese	Patients with T2DM (*n* = 10)	16S sequencing	*Blautia*↓	Inoue et al. (2017)
United States	Patients with T2DM (*n* = 1039)	Bioinformatics analysis	Genera *Allistipes*↓ α‐diversity (Chao1, Shannon, and Simpson) ↓ Family *Bacteroidaceae*, family *Lachanospiraceae*, genera *Bacteroides*, and genera *Facalibacterium*↑	Park et al. (2023)
European	Females with T2DM (*n* = 53)	Metagenome	Five *Clostridium* species↓ Four *Lactobacillus* species↑	Karlsson et al. (2013)
China	Patients with T2DM (Uygurs: *n* = 53, Kazaks: *n* = 53)	16S sequencing	Uygurs: *Erysipelotrichaceae*↓ Kazaks: *Planococcaceae*, and *Coriobacteriaceae*↓ *Veillonellaceae*↑	Wang et al. (2017)
Asians	Patients with T2DM (*n* = 551)	Bioinformatics analysis	ET‐L: *Phocaeicola vulgatus*, *Bacteroides uniformis* , and *Faecalibacterium prausnitzii* ↓ *Escherichia fergusonii* , *Collinsella aerofaciens* , *Streptococcus vestibularis* , and *Bifidobacterium longum* ↑ ET‐P: *Bacteroides koreensis* and *Faecalibacterium prausnitzii* ↓ *Escherichia fergusonii* , *Megasphaera elsdenii* , and *Oscillibacter valericigenes* ↑	Pan et al. (2023)
China	Patients with T2DM and obesity (*n* = 6)	Metagenome	Firmicutes, *Oribacterium*, and *Paenibacillus*↓	Ting Ye et al. (2020)

Abbreviations: ET‐L, *Lachnospiraceae*; ET‐P, *Prevotellaceae*; T2DM, type 2 diabetes mellitus.

### 2.2 Differences in Gut Microbiota Between Animals With T2DM and Normoglycemic Animals

Differences in the composition of gut microbiota between normoglycemic animals and animal models of T2DM have been reported in several studies (Table 2). Animals with T2DM have reduced alpha and beta diversity compared with healthy animals (Ma et al. 2024; Zhao and Fang 2024; Guan et al. 2023; Ohtsu et al. 2019), which is consistent with observations in human studies. Significant alterations were observed in the phyla Firmicutes, Bacteroidetes, and Proteobacteria in diabetic animal models, suggesting that these three phyla may represent core targets associated with gut microbiota dysbiosis in T2DM. Several studies found that the ratio of Firmicutes to Bacteroidetes was reduced in animal models of T2DM (Liu, Ma, et al. 2024; Pan et al. 2023), whereas others found that the ratio of Bacteroidetes to Firmicutes was reduced in animal models of T2DM (Zebiao et al. 2024). The interstudy variability in these findings may be attributed to differences in the age, strain, and dietary patterns of the mice used in the studies. Further, changes at the genus level may not fully represent microbial changes, necessitating more comprehensive investigations at the family, genus, and species levels. In addition, correlation analysis of the faecal gut microbiota in T2DM mice showed that harmful bacteria were significantly increased in the T2DM group compared to the normal group, whereas beneficial bacteria were significantly decreased (Guan et al. 2023). The abundance of probiotics in the gut microbiota of mice decreased with prolonged T2DM (Liu, Yan, et al. 2024). In conclusion, core gut microbiota changes in both humans and animals with T2DM provide potential directions for mechanistic studies. However, species‐specific differences remain. Future research should integrate clinical findings with experimental animal data to further advance T2DM–gut microbiota‐based intervention strategies and clinical translation.

**TABLE 2:** Differences in the gut microbiota between animals with type 2 diabetes mellitus and normoglycemic animals.

Research object	Modelling method	Research method	Compared with the control group, changes in gut microbiota in T2DM animals	References
Four‐week‐old male wild‐type C57BL/6J mice	Injection with 50 mg/kg STZ	16S sequencing	Genera *Brevibacterium*, *Corynebacterium*, and *Facklamia*↑	Ohtsu et al. (2019)
Male C57BL/6 mice	STZ was injected at a dose of 35 mg/kg	16S sequencing	Beta diversity analysis↓ *Bacteroidota, Verrucomicrobiota Akkermansia, Parabacteroides, Lachnospiraceae*_NK4A136_group, and *Bacteroides*↑	Guan et al. (2023)
Six‐week‐old db/db mice	—	16S sequencing	Phylum Bacteroidetes and Deferribacteres↓ Phylum Firmicutes, Proteobacteria, and Fibrobacteres↑	Geurts et al. (2011)
Seven‐week‐old male db/db mice	—	16S sequencing	Firmicutes, Actinobacteria, and the ratio of Firmicutes/Bacteroidetes↓ Bacteroidetes, Epsilon bacteroaeota, Tenericutes, *Prevotellaceae*_UCG‐001, *Alloprevotella, Bacteroides*, and *Prevotellaceae*_NK3B31↑	Pan et al. (2023)
SD rats (180–220 g)	Intraperitoneally injected with 30 mg kg‐1 STZ	16S sequencing	The ratio of Bacteroidetes to Firmicutes↓	Zebiao et al. (2024)
SD male rats aged 6 weeks and weighing 150–190 g	Injected intraperitoneally with 1% streptozotocin at a dose of 30 mg/kg	16S sequencing	Phylum Proteobacteria, class Gammaproteobacteria, family *Enterobacteriaceae* and *unclassified_Firmicutes*↑ Phylum Firmicutes, class Clostridia and Erysipelotrichi, family *unclassified_Clostridiales* and *Micrococcaceae*↓	Han (2024)
Eight‐week‐old male db/db mice	—	16S sequencing	*Bacteroides, Burkholderiaceae*, and *Lactobacillaceae*↑	Guo, Zeng, et al. (2024)
Male C57BL/6 J mice (5 weeks old, 16 ± 2 g)	Intraperitoneal injection of 50 mg/kg STZ	16S sequencing	the ratio of Firmicutes/Bacteroidetes, *Faecalibaculumrodentium*, *uncultured_bacterium_g_Dubosiella*, *uncultured_bacterium_g_Olsenella*, and *Akkermansiamuciniphila*↓ *unclassified_g_Lactobacillus*, *unclassified_g_Cornebacterium*, *Lactobacillus_murinus*, and *Lachnospiraceae_bacterium_10_1*↑	Liu, Ma, et al. (2024)
Sixteen‐week‐old male GK rats	—	16S sequencing	Sobs and ACE indices, *Monoglobus* and *Intestinimonas*↓ *Eubacterium coprostanoligenes* ↑	Zhao and Fang (2024)
Male SD rats (500 ± 20 g, 13 months old)	STZ‐induced	Metagenome	Shannon index, phylum Firmicutes, *Spirochaetes*, and *Proteobacteria*↓ Phylum Bacteroidetes↑	Ma et al. (2024)

Abbreviations: GK, Goto‐Kakizaki; SD, Sprague–Dawley; STZ, streptozotocin.

## 3 Therapeutic Strategies for the Gut Microbiota in T2DM

### 3.1 Probiotics

Studies have demonstrated that *Alistipes finegoldii* can reduce postprandial blood glucose in mice, and *Alistipes indistinctus* exhibits a similar hypoglycemic effect. Moreover, compared to *A. finegoldii* , *A. indistinctus* improves IR and body weight (Takeuchi et al. 2023). *Blautia* shows comparable effects, as both *Blautia producta* and *Blautia wexlerae* effectively prevent obesity, reduce weight gain and fat accumulation, while simultaneously reducing blood glucose and insulin levels, and lowering the homeostasis model assessment of IR (HOMA‐IR) index score compared with mice on a normal diet (Hosomi et al. 2022; Guo, Gao, et al. 2024). *Lactobacillus*, a genus comprising over 200 species, refers to a group of bacteria that metabolise sugars such as glucose into lactic acid. In both clinical and animal studies, different *Lactobacillus* strains have been found to reduce blood glucose, haemoglobin A1c (HbA1c), and cholesterol levels, and improve IR (Zhong et al. 2024; Yao et al. 2019; Wu, Zhang, et al. 2021; Dang et al. 2018; Hsieh et al. 2018). *Lactobacillus* upregulates the expression of glucagon‐like peptide‐1 (GLP‐1) and interleukin (IL)‐10 in patients with obesity or T2DM and suppresses lipid accumulation in adipocytes (Kim et al. 2019; Alard et al. 2021). Furthermore, *Lactobacillus fermentum* *MCC2760* increased the expression of glucose transporter type 4 (GLUT4), GLP‐1, and zonula occludens‐1 (ZO‐1), thereby improving glucose tolerance in mice fed a high‐fat diet (Archer et al. 2021). *Lactobacillus paracasei* *L14* and *Lactobacillus gasseri* *CKCC1913* ameliorated T2DM‐related metabolic health by reducing oxidative stress and inflammatory responses (Zeng et al. 2023). *Lactobacillus plantarum* *HAC01* and *L. paracasei IMC 502* alleviated hyperglycaemia by preserving pancreatic β‐cell function and modulating glucose metabolism (Lee, Lee, et al. 2021; Gu et al. 2023). *Lactobacillus salivarius* *AP‐32* and *Lactobacillus reuteri* *GL‐104* rapidly consumed hexoses and downregulated sodium glucose co‐transporter 1 (SGLT1) and glucose transporter 5 (GLUT5) in Caco‐2 colorectal adenocarcinoma‐derived epithelial cells, suggesting their potential to significantly inhibit intestinal sugar uptake (Hsieh et al. 2020). A groundbreaking study revealed that daily administration of live cells from an *Akkermansia muciniphila* ‐type strain (10^8^ CFU/day) to mice for 4 weeks counteracted the adverse metabolic effects associated with a high‐fat diet (Everard et al. 2013). Furthermore, the administration of *A. muciniphila* to high‐fat diet‐fed mice improved glucose tolerance, which was consistent with the effects observed in metformin‐treated high‐fat diet‐fed mice. Moreover, *A. muciniphila* enhances the intestinal barrier by upregulating the expression of tight junction proteins (Lee, Chae, et al. 2021). *A. muciniphila* secretes the P9 protein, which binds to its ligand intracellular adhesion molecule‐2 and subsequently activates the GLP‐1 receptor and IL‐6 signalling pathways. This process enhanced thermogenesis, thereby ameliorating high‐fat diet‐induced obesity and improving glucose homeostasis in mice (Yoon et al. 2021). The outer membrane protein Amuc_1100 of *A. muciniphila* also promotes GLP‐1 secretion, although its effect is considerably weaker than that of the P9 protein (Yoon et al. 2021; He et al. 2024). *F. prausnitzii* , the most abundant bacterial strain in humans, also plays a crucial role in maintaining intestinal health (Zixi et al. 2021). The bacterium produces butyric acid, which is associated with T2DM. Transplantation of *F. prausnitzii* has been used as an intervention strategy to treat gut microbiota dysbiosis, which is linked to autoimmune diseases and prediabetic inflammation (Ganesan et al. 2018). Furthermore, on the basis of assessments of lipid‐regulating enzyme activities and pro‐inflammatory cytokine levels, *F. prausnitzii* not only alleviates inflammation in adipose tissue and the liver, but also improves hepatic steatosis by suppressing the activity of hepatic lipogenic enzymes (Rossi et al. 2016; Xuan et al. 2023). *F. prausnitzii* contributes to the maintenance of intestinal immune homeostasis and attenuates inflammation by inducing IL‐10 production and modulating T cell responses (Rossi et al. 2016; Alameddine et al. 2019). Additionally, butyrate produced by *F. prausnitzii* maintains the T helper 17/T regulatory cell balance through inhibition of histone deacetylase (HDAC) 1, further mitigating inflammation (Zhou et al. 2018). Cardinali et al. (2020) reported that two patients with refractory T2DM who were treated with conventional therapy alongside *Bacillus subtilis* Natto DG101 showed a significant decrease in blood glucose, HbA1c, and insulin levels, with values approaching the normal range. Furthermore, throughout the treatment period, patients maintained normal hepatic and renal functions without any adverse effects (Cardinali et al. 2020). *Alistipes*, *Blautia*, *Lactobacillus*, *F. prausnitzii* , and *A. muciniphila* improved glucose and lipid metabolism to varying degrees. However, specific therapeutic effects differ depending on the strain. These differences reflect the strain‐specific properties of probiotics. Distinct strains exhibit unique metabolic functions and mechanisms of action and are also associated with experimental design factors, such as animal strain and diet. Current research remains predominantly on the basis of animal studies; however, future efforts should strengthen the continuum from “strain screening–human trials–mechanistic investigation” to promote the practical application of probiotics in the management of T2DM (Table 3).

**TABLE 3:** Comparisons of efficacy, safety, and applicability of probiotic, prebiotic, and FMT in T2DM management.

Strategies	Efficacy	Safety	Applicability	References
**Probiotics**				
*Alistipes indistinctus*	PPG, HOMA‐IR, and body weight↓ Hepatic ectopic triglyceride accumulation and impaired glucose tolerance↑	—	C57BL6/N male mice aged 6 weeks with Quick Fat (CLEA Japan)	Takeuchi et al. (2023)
*Alistipes finegoldii*	PPG↓	—	C57BL6/N male mice aged 6 weeks with Quick Fat (CLEA Japan)	Takeuchi et al. (2023)
*Blautia producta*	Blood glucose, TC, and LDL‐c↓	A type strain of Blautia recognised as safe for use in food and medicine	C57BL/6J male mice with a high‐fat diet	Guo, Gao, et al. (2024)
*Blautia wexlerae*	Blood glucose, insulin levels, HOMA‐IR, body weight and fat accumulation↓	—	Male C57BL/6 mice (age, 4 weeks) with a high‐fat diet	Hosomi et al. (2022)
*Lactobacillus paracasei N1115*	FBG, FI, HOMA‐IR, TC, TG↓	—	C57BL/6 mice with a high‐fat diet	Yao et al. (2019)
*Lactobacillus plantarum*	FBG, HbA1c, HOMA‐IR, TC, TG, LDL‐c↓ HDL‐c↑	—	Patients with T2DM and prediabetes	Zhong et al. (2024)
*Akkermansia muciniphila*	FBG, HOMA‐IR, body weight↓ Body composition (i.e., fat mass/lean mass ratio)↑	—	Male C57BL/6 mice with a high‐fat diet	Everard et al. (2013)
*Bacillus subtilis natto DG101*	Blood glucose、HbA1c and insulin levels↓	The patients had normal liver and renal functions, and no adverse events were observed	Patients with T2DM	Cardinali et al. (2020)
**Prebiotics**				
Resistant dextrin	FBG, FI, HOMA‐IR, TC and LDL‐c↓	—	Four‐week‐old male C57BL/6 mice with a high‐fat‐high‐fructose diet	Hu et al. (2020)
Garlic	FI, HOMA‐IR, TG, TC and LDL‐c↓	It does not cause any damage to the liver and may alleviate the burden on the liver caused by a high‐fat diet	Male C57BL/6N mice (5 weeks of age) with a high‐fat diet	Chen et al. (2019)
Inulin oligofructose	FBG and blood lipid levels↓ Oral glucose tolerance and insulin sensitivity↑	—	Male Wistar rats 8–9 weeks old with a high‐carbohydrate, high‐fat diet	Kumar et al. (2016)
Inulin	FBG, HbA1c and blood lipid levels↓	—	Female patients with T2DM	Dehghan et al. (2016)
Acorn‐ and sago‐ derived prebiotics	Glucose clearance and insulin sensitivity↑	—	C57BL/6J mice (age 8–10 weeks) with a high‐fat diet	Ahmadi et al. (2019)
FOS	FBG, FI, HOMA‐IR, TG and TC↓	—	C57BL/6 mice with a high‐fat diet	Yao et al. (2019)
GOS	Body weight, HOMA‐IR and blood lipid levels↓		Male C57BL/6OlaHsd mice with a Western‐Type diet	Mistry et al. (2020)
**FMT**				
Mice	Glucose clearance↑		Male C57BL/6 mice with a high‐fat diet	Zhang et al. (2023)
Mice	Body weight, HbA1c, HOMA‐IR, TG, and LDL‐c↓ Glucose clearance and HDL‐c↑	—	Male 5‐year‐old ZDF rats	Lijing et al. (2020)
Healthy individuals from Chinese Kazaks	Blood glucose, TC, TG, and LDL‐c↓ HDL‐c↑	—	Eight‐week‐old male db/db mice	Wang et al. (2017)
Kunming mice	FBG, HbA1c and HOMA‐IR↓ HOMA‐IS and HOMA‐β↑	—	Kunming mice with a high‐fat diet and combined with a single intraperitoneal injection of streptozotocin (100 mg/kg)	Wang and Bai (2020)
Healthy donor	BMI, FBG, PPG, HbA1c and HOMA‐IR↓ HOMA‐β↑	No AEs were observed, and no cases of hypoglycaemia or dyslipidaemia occurred	Adult T2DM patient	Wu, Zhang, et al. (2023)
Healthy donor	FBG and TG↓	No serious AEs	Patients with hyperglycaemia	Wu, Li, et al. (2022)
Healthy donor	BMI, FBG, and blood lipid levels↓	No serious AEs	Patients with metabolic syndrome	Wu, Lu, et al. (2022)
Healthy donor	TCSS score, VAS score, and anxiety↓ Sleep quality↑	No serious AEs	Distal symmetric polyneuropathy in patients with diabetes	Yang et al. (2023)

Abbreviations: AEs, adverse events; BMI, body mass index; FBG, fasting blood glucose; FI, fasting insulin; FMT, faecal microbiota transplantation; FOS, fructooligosaccharides; GOS, galactooligosaccharides; HbA1c, glycated haemoglobin; HDL‐c, high‐density lipoprotein cholesterol; HOMA‐β, homeostasis model assessment‐β; HOMA‐IR, homeostasis model assessment of insulin resistance; HOMA‐IS, homeostasis model assessment of insulin sensitivity; LDL‐c, low‐density lipoprotein cholesterol; PPG, postprandial blood glucose; T2DM, type 2 diabetes mellitus; TC, total cholesterol; TCSS, Toronto clinical scoring system; TG, triglyceride; VAS, visual analog scale.

### 3.2 Prebiotics

Administration of prebiotics has been shown to decrease excessive food intake and dysglycaemia in db/db mice, which carry a point mutation in the leptin receptor gene, causing leptin receptor deficiency and defective leptin signalling (de Cossío et al. 2017). Resistant dextrin derived from wheat and corn starch enhances lipid and glucose metabolism in insulin‐resistant mice by increasing the abundance of *Akkermansia* and *Prevotella* (Hu et al. 2020). Garlic, which contains prebiotics, fructans, antimicrobial compounds, and organosulfur compounds, ameliorated dyslipidaemia and IR in high‐fat diet‐fed mice. Additionally, garlic increases the α‐diversity of the gut microbiota, enhances the abundance of *Lachnospiraceae*, and decreases *Prevotella* (Chen et al. 2019). Animal studies have demonstrated that inulin improves high‐fat diet‐induced generalised obesity and reduces fasting blood glucose levels, oral glucose tolerance, and IR in rats (Kumar et al. 2016). Similarly, clinical studies have indicated that inulin provides multiple benefits including improvements in blood glucose levels, lipid indices, and immune indices in patients with T2DM (Dehghan et al. 2016). Mice fed acorn‐ and sago‐derived prebiotics showed greater improvement in high‐fat diet‐induced glucose intolerance and IR than those fed inulin (Ahmadi et al. 2019). Fructooligosaccharides (FOS) effectively reduced fasting glucose and insulin levels to baseline levels in mice with nonalcoholic fatty liver disease, thereby significantly alleviating IR. An intraperitoneal glucose tolerance test demonstrated that the effects of FOS on glucose metabolism were observable within 30 min and persisted for at least 120 min (Yao et al. 2019). Feeding with galactooligosaccharides (GOS) resulted in significantly elevated levels of Actinobacteria, particularly *Bifidobacterium* and *Parvibacter*, as well as Betaproteobacteria: *Parasutterella*, *Akkermansia*, and an uncultured genus within the family *Erysipelotrichaceae*. Additionally, dietary fiber supplementation such as with GOS was associated with a significant reduction in Firmicutes taxa, specifically within Clostridia, affecting families such as *Lachnospiraceae*, *Ruminococcaceae*, and *Peptostreptococcaceae*, together with genera including *Olsenella*, *Alistipes*, *Faecalibaculum*, and *Bilophila* (Mistry et al. 2020). GOS also ameliorated the effects of metabolic syndrome in patients with weight gain, dyslipidaemia, and insulin sensitivity, suggesting that GOS has considerable therapeutic potential for glucose metabolism. Consequently, prebiotics play a vital role in enhancing blood glucose control, improving insulin sensitivity, and modulating the composition of gut microbiota in patients with T2DM. Most prebiotics exert beneficial effects on lipid metabolism. Additionally, certain natural prebiotics such as those derived from garlic have hepatoprotective effects (Table 3).

### 3.3 Probiotic Combinations and Synbiotics

The effects of combinations of probiotics and synbiotics (prebiotics and probiotics) on blood glucose levels have been investigated. In obese mice treated with a mixture of *Lactobacillus rhamnosus LMG S‐28148* and *Bifidobacterium animalis subsp. lactis LMG P‐28149*, fasting blood glucose and insulin levels were significantly reduced compared to those in mice fed a high‐fat diet (Alard et al. 2016). Additionally, yogurt enriched with *Lactobacillus* and *Bifidobacterium* lowers fasting blood glucose, fasting insulin, and IR levels in T2DM mice over 12 weeks. This dietary intervention also resulted in a significant decrease in the relative liver weight and liver triglyceride levels. Yogurt consumption contributes to the maintenance of glucose homeostasis and insulin sensitivity while improving hepatic steatosis and function (Daniel et al. 2022). Probioglu, a multi‐strain probiotic supplement containing *Lactobacillus salivarius subsp. salicinius AP‐32*, *Lactobacillus johnsonii MH‐68*, *L. reuteri GL‐104*, and *B. animalis subsp. lactis CP‐9*, significantly improves glucose tolerance and reduces blood glucose, insulin, and IR levels in mice with diabetes (Pei Shan et al. 2021). Similarly, gavage administration of FMT containing *Lactobacillus* spp. and *Bifidobacterium bifidum* to T2DM mice over a 6‐week period exhibited a comparable glucose‐lowering effect, slight reduction in endotoxaemia, and restoration of normal tissue morphology in the colonic mucus layer and hepatic lobules (Wang, Yang, et al. 2022). Clinical studies have shown that the administration of synbiotics improves HbA1c levels, BMI, and microalbuminuria in patients with T2DM (Ebrahimi et al. 2017). In comparison to baseline levels, the quantities of *Bifidobacterium* and total *Lactobacillus* in the faeces of patients exhibited increases in their relative abundance, particularly for *Bifidobacterium* species such as *B. adolescentis* and *B. pseudocatenulatum* , together with increases in the concentrations of acetic and butyric acids (Kanazawa et al. 2021). Among the synbiotic‐treated patients with T2DM, the most notable difference in the microbiome before and after treatment was the abundance of *Levilactobacillus brevis*. Additionally, a decrease in HbA1c levels was observed in patients with *L. brevis* ^+^, whereas an increase was noted in patients with *L. brevis* ^−^ (Horvath et al. 2020). Consequently, the combined use of probiotics and synbiotics leads to improvements in blood glucose levels and insulin sensitivity, and partial enhancement of the intestinal environment in patients with T2DM. One study indicated that the combination of *L. paracasei* N1115 and FOS was more effective in reducing IR than *L. paracasei* N1115 or FOS alone (Yao et al. 2019). Synbiotic treatment demonstrated a significant alleviating effect on fasting blood glucose and IR in T2DM, and the combined application proved to be superior to individual treatments. However, the specific effects warrant further investigation.

### 3.4 FMT

#### 3.4.1 FMT for Management of T2DM

Several studies have investigated the role of FMT in diabetes (Table 3). Both short‐ and long‐term FMT have demonstrated therapeutic effects. Four weeks after the FMT intervention, glucose tolerance significantly improved in mice with diet‐induced obesity (Zhang et al. 2023). Our previous study found that WMT resulted in short‐ and mid‐term improvements in fasting blood glucose levels in hyperglycemic patients without disrupting glycemic responses in normoglycemic individuals (Wu, Li, et al. 2022). A 4‐year follow‐up study demonstrated that FMT significantly improved waist circumference, total body fat, metabolic syndrome severity score, and systemic inflammation, and increased high density‐lipoprotein cholesterol levels (Wilson et al. 2025). Similarly, another long‐term clinical efficacy study observed comparable effects, with multi‐session WMT yielding superior outcomes (Lin et al. 2024). In addition to reducing fasting blood glucose, postprandial blood glucose, and glycated haemoglobin and improving glucose tolerance and insulin sensitivity (Wang and Bai 2020; Wu, Zhang, et al. 2023; Lijing et al. 2020; Aron‐Wisnewsky et al. 2019), we discovered that FMT ameliorates inflammation and atherosclerotic cardiovascular disease risk classification in hyperglycemic patients (Wu, Li, et al. 2022). Moreover, the degree of improvement with FMT was significantly greater than that in T2DM patients treated with metformin (Wu, Zhang, et al. 2023). FMT also plays an important role in distal symmetric polyneuropathy in patients with diabetes, which is a complication of diabetes. Participants who underwent FMT experienced an approximately 35% reduction in neuropathic pain, whereas those who did not receive FMT experienced only about a 5% reduction in pain. FMT induced early and significant alterations in the gut microbiota of distal symmetric polyneuropathy in patients with diabetes, accompanied by marked and sustained improvements in neurological signs and symptoms (Yang et al. 2023). Furthermore, FMT can modify the structure of the gut microbiota and the metabolic profiles of patients (Lijing et al. 2020; Wu, Lu, et al. 2022) and restore the homogeneity of the gut microbiota in T2DM mice (Wang and Bai 2020). Zhang et al. (Zhang, Li, et al. 2020) indicated that in db/db mice treated with FMT, the abundance of *Desulfovibrio* and *Clostridium coccoides* in the gut was significantly reduced, whereas the level of *A. muciniphila* in the faeces was elevated. Another study found that the genera *Lactobacillus*, *Clostridium*, and *Rothia* were negatively correlated with all glycemic parameters (Lijing et al. 2020).

In summary, FMT improved the blood glucose levels in obese animal models, obese patients, a range of T2DM animal models, and patients with T2DM. Notably, the efficacy of FMT in improving the blood glucose levels was comparable to that of metformin. These findings underscore the clinical significance of FMT. This procedure can modify the composition of the gut microbiota, demonstrating beneficial effects in the treatment of T2DM, thus warranting further investigation into the roles of specific bacteria. However, more comprehensive clinical studies of FMT for the management of diabetic complications are required.

#### 3.4.2 Advantages of WMT

As early as the Eastern Jin Dynasty, more than 1700 years ago, there was a reference to the use of faecal clearing for treating diseases, as documented in the *Handbook of Prescriptions for Emergencies* compiled by Ge Hong (Ge 2000). The use of human faeces to treat severe diarrhoea and food poisoning was also noted in the Compendium of Materia Medica compiled by Li Shizhen during the Ming Dynasty (Li 2011). In 1958, Dr. Eiseman, a surgeon at the University of Colorado School of Medicine in the United States, used human faecal‐clearing enemas to treat pseudomembranous enterocolitis and achieved favourable results (Eiseman et al. 1958). The in‐depth study of FMT has demonstrated good results in the treatment of *C. difficile* infection, autism, ulcerative colitis, and other diseases (Pan et al. 2022; Schwan et al. 1983; Zhang et al. 2013; Zhang et al. 2012; Surawicz et al. 2013). Safety risks associated with FMT disease treatment remain. A previous report indicated that two patients developed ultra‐broad‐spectrum β‐lactamase bacteraemia following FMT, resulting in the death of one patient (DeFilipp et al. 2019). The challenging FMT preparation process, which involves contact with faeces, poses psychological barriers to its acceptance by physicians, patients, and donors, thereby limiting its broad public acceptance (Brandt 2012; Leslie et al. 2017). In contrast, WMT uses an intelligent faecal bacterial separation device (GenFMTer, Nanjing, China) that automates the preparation process and incorporates multiple washing steps. This method effectively removes undigested food residues, fungi, parasite eggs, and certain pro‐inflammatory metabolites, facilitating the production of bacterial fluids with controllable quality and enhanced safety (Shi 2020). A multifactorial analysis demonstrated that WMT significantly reduced adverse events from 38.7% to 14.4% in patients with ulcerative colitis (Ding et al. 2019). Furthermore, another study reported a reduction in the incidence of adverse events to 8.7% with WMT compared to 21.7% with FMT in patients with Crohn's disease (Wang et al. 2018). Notably, the efficacy of WMT is comparable to that of FMT in the treatment of diseases (Ding et al. 2019; Wang et al. 2018). Thus, WMT has the advantages of enhanced safety, precision, and quality control compared with conventional FMT.

#### 3.4.3 Factors Affecting Treatment Efficacy

**FIGURE 1:**
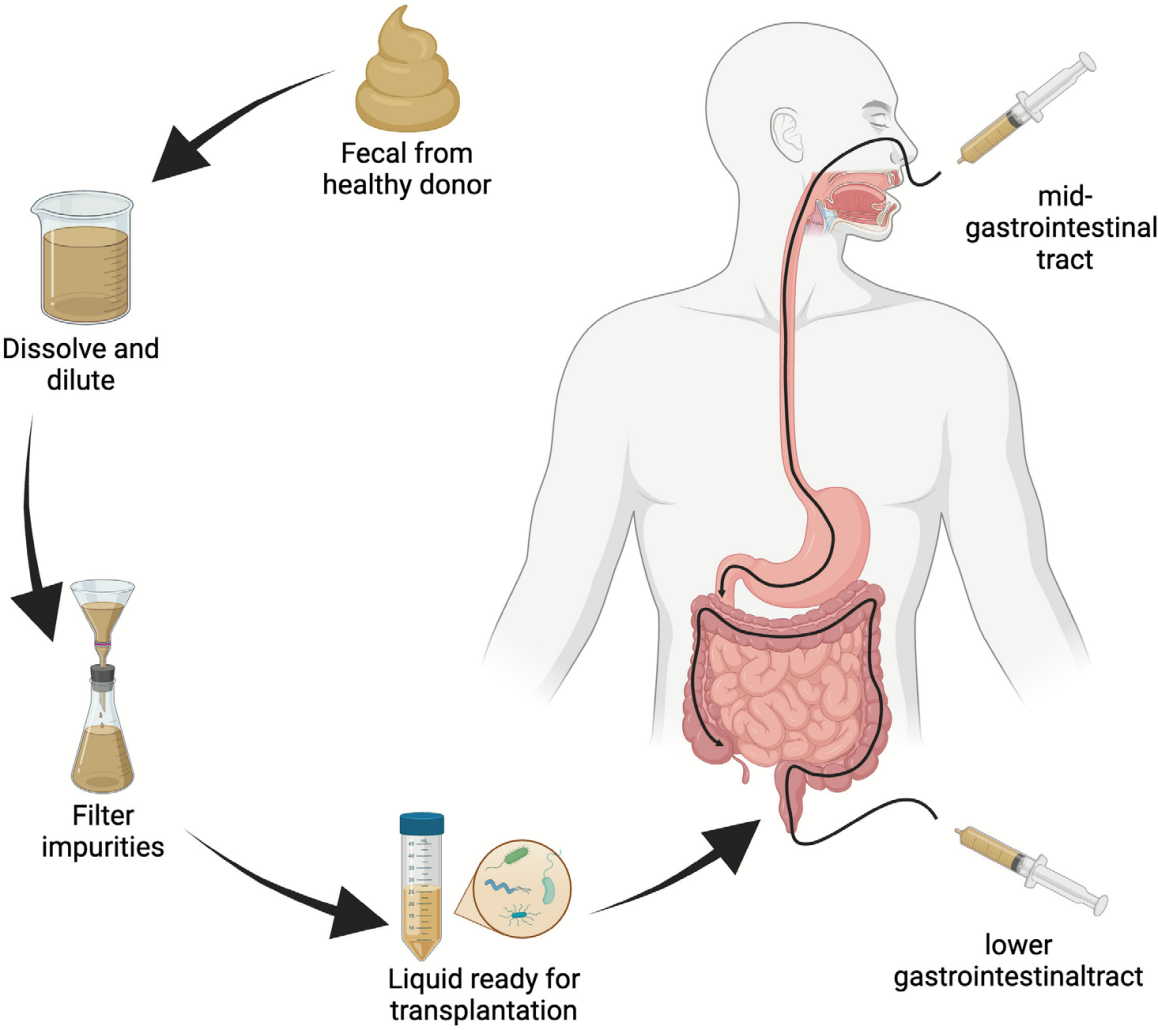
The faecal microbiota transplantation process. Donor faecal samples were dissolved, diluted, filtered, and centrifuged to prepare washed microbiota suspensions for transplantation. The faecal microbiota suspension is administered to the patient via the mid‐gastrointestinal tract or lower gastrointestinal tract, depending on the specific condition and preferences of each patient.

Faecal bacteria from patients with T2DM have been shown to cause abnormalities in glucose and lipid metabolism in normoglycemic mice (Hu 2016), impairing insulin tolerance and oral glucose tolerance while altering the composition of the gut microbiota, leading to a significant reduction in *Prevotella* at the genus level (Wang, Wang, et al. 2022). Furthermore, the gut microbiota of distal symmetric polyneuropathy in patients with diabetes induces a more severe peripheral neuropathy phenotype in db/db mice (Yang et al. 2023). In a clinical trial, obese patients who received FMT from healthy, lean individuals demonstrated positive changes in insulin sensitivity (Aron‐Wisnewsky et al. 2019). Notably, improvements in insulin sensitivity in patients with metabolic syndrome were observed exclusively in the group who received FMT from lean donors or their own gut microbiota (Vrieze et al. 2012). Similarly, impaired glucose tolerance and IR improved in individuals with exercise sensitivity to insulin and homeostatic glucose responses, as well as in patients with normal glucose tolerance (Zhang, Li, et al. 2020; Liu et al. 2020). Thus, donor health status plays a crucial role in determining FMT outcomes. After FMT, the similarity of the gut microbiota between the patient and the corresponding graft was significantly reduced; however, the composition of the gut microbiota of the patient remained closer to its baseline than that of the graft (Yang et al. 2023). Additionally, one study found that among FMT‐treated patients with T2DM, the family *Rikenellaceae* and genus *Anaerotruncus* (class Ruminalococcaceae) were more abundant in the gut microbiota of patients with good treatment outcomes than in those with poor outcomes. These findings suggest that *Rikenellaceae* and *Anaerotruncus* may serve as potential biomarkers in patients with T2DM undergoing FMT (Ding et al. 2022). Therefore, the status of the patient's gut microbiota is a critical factor influencing FMT treatment efficacy and warrants further investigation because of the study's small sample size. Some studies have indicated that when the intestinal type is matched between the donor and recipient, changes in the gut microbiota of patients are more pronounced after bacterial transplantation and the improvement in various patient indicators is more significant. The gut microbiota similarity between donors and recipients is important in enhancing FMT therapeutic outcomes. The effects of host and microbiome parameters on the efficacy of FMT warrant further investigation. Given the unique composition of the gut microbiota in patients, the development of personalised microbiota‐based therapies may improve the outcomes of patients with diabetes. A recent phase II study demonstrated promising results using a mixture of eight commensal *Clostridia* strains for the prevention of recurrent *C. difficile* infections, suggesting that identifying the core commensal microbiota responsible for the beneficial effects of FMT may enable the development of a safe and effective alternative to this therapy (Louie et al. 2023; Zhou et al. 2019; Wu, Zuo, et al. 2021). The use of specific bacterial consortia or genetically engineered bacteria to enhance the therapeutic efficacy of FMT is promising; however, several technical challenges remain (Ratiner et al. 2024). For instance, certain engineered strains, such as *Escherichia coli* *strains MG1655* and Nissle 1917, exhibit limited capacity for host colonisation (Russell et al. 2022). Editing native strains may overcome this challenge (Russell et al. 2022); however, further clinical trials are required for validation. FMT methods include procedures targeting the mid‐gastrointestinal and lower gastrointestinal tracts (Figure 1). The lower gastrointestinal tract includes transgastroscopic transendoscopic tube (TET) placement, whereas the mid‐gastrointestinal tract includes transgastroscopic TET placement and manual spiral nasojejunal tube placement. At the time of initial TET placement, patients showed a preference for transgastroscopic TET placement, although lower gastrointestinal tract TET placement was superior to mid‐gastrointestinal tract TET placement (Zhong et al. 2023). This superiority may be attributed to the location specificity of gut microorganisms, which tend to colonise similar gut regions (Na et al. 2020), and to bowel preparation before lower gastrointestinal tract TET placement, which facilitates microbial colonisation (Zhong et al. 2023). Furthermore, another study demonstrated no significant difference in overall comfort scores or the incidence of common discomfort between gastroscopic TET placement and manual spiral nasojejunal tube placement. However, manual spiral nasojejunal tube placement led to a more pronounced improvement in clinical outcomes than gastroscopic TET placement (Zheng et al. 2024). Therefore, the therapeutic effects of different tube placement methods on colony transplantation may vary. Studies have shown that multiple WMT sessions conducted over the course of 1 year are more effective than single‐ or double‐session treatments in improving the lipid profiles of obese patients and reducing the risk of coronary atherosclerotic cardiovascular disease (Lin et al. 2024). Consequently, FMT offers a safe and effective long‐term clinical treatment option for patients receiving multiple courses of consolidation therapy that enhances the colonisation of normal gut microbiota.

### 3.5 The Potential Harmful Effects of Gut Microbiota Modulators

Despite microecological modulator therapy's demonstrated therapeutic benefits, emerging evidence has identified potential adverse effects associated with its clinical application. Documented complications include systemic infections, deleterious metabolic activities, excessive immune activation in predisposed individuals, horizontal gene transfer, and gastrointestinal disturbances (Doron and Snydman 2015). These adverse outcomes are particularly prevalent in immunocompromised patients, premature neonates, individuals with short bowel syndrome, those with central venous catheters, and patients with valvular heart disease (Kumar et al. 2022). One clinical report documented the case of a 74‐year‐old diabetic patient who developed pneumonia and a hepatic abscess after 4 months of *L. rhamnosus GG* supplementation (Foberg et al. 1986). The risk profile is elevated in specific patient populations as evidenced by a randomised, double‐blind, vehicle‐controlled trial investigating a synbiotic formulation (comprising *Lactobacillus acidophilus* , *Lactobacillus casei* , *L. salivarius* , *Lactococcus lactis* , *B. bifidum* , *B. lactis* , cornstarch, and maltodextrins) in patients with severe acute pancreatitis. Although no significant differences were observed in the primary endpoints, the synbiotic group exhibited significantly higher mortality rates than the vehicle group (Besselink et al. 2008). Furthermore, Young et al. (Young and Vanderhoof 2004) reported probiotic‐associated bacterial sepsis in paediatric populations, with two premature infants with short gut syndrome developing *Lactobacillus* bacteraemia after probiotic supplementation. Additional concerns have been raised regarding the potential transfer of antibiotic resistance genes from probiotic strains to pathogenic bacteria, which may exacerbate disease progression (Gueimonde et al. 2013). These findings underscore the necessity for a scientifically rigorous approach to microecological modulator applications, emphasising the importance of comprehensive adverse event documentation. Future research should prioritise large‐scale, high‐quality, strain‐specific randomised controlled trials to establish safety profiles and provide evidence‐based clinical recommendations.

## 4 Mechanisms of Gut Microbiota Modulators Acting on T2DM

### 4.1 Intestinal Barrier Alterations

**FIGURE 2:**
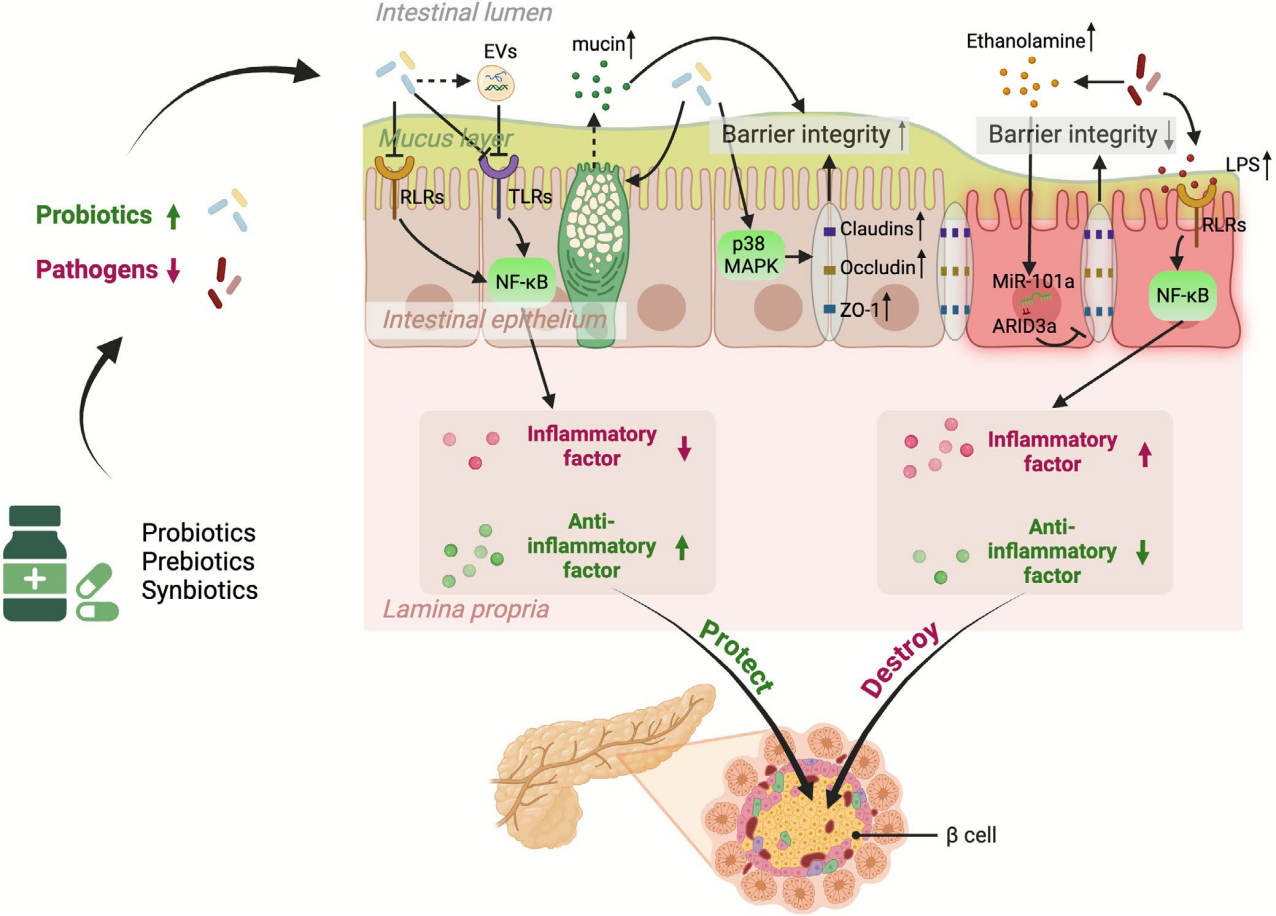
The primary mechanisms through which gut microbiota modulators regulate metabolism, intestinal barrier function, and inflammation in type 2 diabetes mellitus (T2DM) are multifaceted. Gut microbiota modulators directly ameliorate T2DM by modulating the abundance of beneficial and harmful bacteria. An increase in beneficial bacteria enhances the intestinal barrier by stimulating mucin secretion in goblet cells and promoting the expression of tight junction proteins via the p38 MAPK pathway. Concurrently, signaling pathways such as RLRs and TLRs are inhibited, which in turn suppresses the inflammatory response. EVs, Extracellular vesicles; LPS, Lipopolysaccharide; MAPK, mitogen‐activated protein kinase; RLRs, retinoic acid‐inducible gene‐I‐like receptors; TLRs, Toll‐like receptors; ZO‐1, zonula occludens‐1.

When the intestinal mucosal barrier is impaired, harmful substances can penetrate the tissues and bloodstream beneath the intestinal mucosa, triggering inflammatory and immune responses that ultimately impair insulin signalling and glucose metabolism (Khan et al. 2020; Sato et al. 2014) (Figure 2). Gut bacteria can translocate into the bloodstream and play a significant role in the development of IR in patients with T2DM. Consumption of *L. casei* strain in Shirota‐fermented milk has been shown to reduce bacterial translocation and alter the gut microbiota in patients with T2DM (Hsieh et al. 2018). Another previous study demonstrated that the gut microbiota of obese individuals could alter the permeability of the intestinal mucosa and increase the secretion of lipopolysaccharides (LPS), potentially leading to chronic low‐grade inflammation, IR, and T2DM (Diamant et al. 2011). T2DM itself may also influence the composition of gut microbiota and intestinal barrier function. Conversely, gut dysbiosis and disruption of the intestinal barrier can induce IR and chronic inflammation, thereby promoting the progression of T2DM (Cani 2017; Tilg and Moschen 2014). Therefore, enhancing the structure of the gut microbiota could partially restore the function of the gut barrier, reduce chronic inflammation, and improve energy metabolism, thus representing a novel approach for diabetes treatment (Xie et al. 2021; Iatcu et al. 2021). Probiotics can modify the composition of the microbiota to favour *Bifidobacterium* spp., which assist in maintaining healthy gut barrier function, thereby mitigating systemic hormonal and inflammatory dysregulation. Consequently, these improvements may lead to reduced food intake and improved diabetes management (de Cossío et al. 2017; Frost et al. 2014). In addition, extracellular vesicles (EVs) derived from the gut microbiota and probiotics encapsulate a wide range of bioactive molecules, including proteins and nucleic acids, which can propagate over short or long distances. This allows them to modulate important biological functions and has a significant impact on host health. EVs derived from specific bacteria induce various physiological responses. EVs regulate inflammation, metabolism, and intestinal permeability, potentially playing a role in the onset, development, and management of obesity and diabetes (Díez‐Sainz et al. 2022). For instance, *A. muciniphila* ‐derived EVs have been shown to enhance the expression of tight junction genes and downregulate the expression of toll‐like receptor (TLR) genes, suggesting a potential role of *A. muciniphila* ‐derived EVs in enhancing intestinal barrier permeability and reducing inflammation (Ashrafian et al. 2019). Microbial metabolites play significant roles in glucose metabolism. Accumulation of the harmful metabolite ethanolamine in the gut can increase intestinal permeability. Restoring the ethanolamine metabolic activity of the gut microbiota using a novel probiotic therapy reduced elevated intestinal permeability, inflammation, and abnormal glucose metabolism by inhibiting miR‐101a‐3p and thereby enhancing ZO‐1 mRNA stability (Mishra et al. 2023). Intestinal mucins, zonulins, occludins, claudins, and ZO maintain the integrity and function of the intestinal mucosal barrier. Studies have found that potential probiotic cultures can normalise TLR‐4 receptors and markers related to intestinal barrier integrity (ZO‐1) and insulin sensitivity (GLUT‐4, GLP‐1, and adiponectin) (Archer et al. 2021). Isomaltodextrin reduces plasma LPS concentration, decreases macrophage infiltration into adipocytes, and increases the expression of mucins 2 and 4 and the tight junction protein claudin 4, thereby ameliorating IR (Hann et al. 2019). Synbiotics improve intestinal barrier function and tissue integrity by upregulating the expression of occludin‐1 and claudin‐1 via the p38 MAPK pathway (Yao et al. 2019). In patients with T2DM, the combined probiotic treatment significantly reduced serum zonulin levels, and the extent of this reduction was associated with *L. brevis* (Horvath et al. 2020).

Disturbances in the gut microbiota of patients with T2DM are closely linked to intestinal barrier disruption. The administration of microecological agents has been shown to enhance the abundance of beneficial bacteria in these patients. These interventions regulate glucose metabolism through several mechanisms, including reducing bacterial translocation, lowering the endogenous LPS level, decreasing the zonulin level, and promoting the expression of mucin and tight junction proteins. Additionally, these agents modulate the p38 MAPK pathway and ARID3a/miR‐101a/ZO‐1 axis, ultimately improving intestinal permeability.

### 4.2 Immunity and Inflammation Regulation

T2DM is a chronic inflammatory disease characterised by elevated levels of inflammatory factors, including tumour necrosis factor (TNF). This inflammatory state can lead to IR, which subsequently exacerbates inflammatory conditions (Figure 2). Insulin has been shown to exert anti‐inflammatory effects at the cellular and molecular levels in vitro and in vivo. However, disruption of insulin signalling can hinder these anti‐inflammatory effects (Dandona 2004). Although diabetes induces islet cell apoptosis, FMT has been shown to significantly restore islet β‐cell function in T2DM mice, thereby improving IR and mitigating islet damage. This effect is mediated by a reduction in pro‐inflammatory cytokine secretion and an increase in anti‐inflammatory cytokine secretion (Wang and Bai 2020). Accumulating evidence indicates that gut microbiota play a significant role in the pathogenesis of low‐grade inflammatory diseases. Gut microbes influence the mucosal immune system by regulating the differentiation and expansion of various T cell types, thereby alleviating T2DM symptoms (Furusawa et al. 2013). Metabolic dysregulation typically occurs prior to the onset of β‐cell autoimmunity and diabetes. These findings suggest that interventions in metabolism or immune regulation before the onset of autoimmunity may represent potential strategies for the prevention of T2DM (Orešič et al. 2008). A decrease in the abundance of probiotics, such as *Bacteroides* and *Faecalibacterium*, may trigger inflammatory responses in T2DM by activating the retinoic acid‐inducible gene‐I‐like receptor signalling pathway, which in turn activates the downstream NF‐κB signalling pathway (Liu, Yan, et al. 2024). Inulin significantly increases serum IL‐4 levels in patients with T2DM. IL‐4 is a potent anti‐inflammatory cytokine that inhibits the production of inflammatory mediators, such as TNF‐α, IL‐1, and prostaglandin E2, by human monocytes and macrophages. Furthermore, IL‐4 enhances the function of regulatory T helper 2 cells while inhibiting T helper 1 cell‐mediated destruction of β islet cells, thereby preventing insulin‐dependent diabetes (Dehghan et al. 2016). Additionally, reductions in IL‐12 and interferon‐gamma following probiotic treatment further corroborate these beneficial effects, with the serum IL‐12 level being associated with peripheral IR and pancreatic β‐cell dysfunction in patients with T2DM (Dehghan et al. 2016). In an animal model of streptozotocin‐induced diabetes, administration of Probioglu was shown to increase β‐cell mass and stabilise the blood glucose level, while concurrently decreasing levels of the pro‐inflammatory cytokines TNF‐α, IL‐6, and IL‐1β (Pei Shan et al. 2021). Furthermore, the administration of fermented *L. reuteri* reduced inflammation in the liver, muscle, and adipose tissue of diabetic rats (Archer et al. 2021). Endocrine activity in adipose tissue plays a crucial role in regulating glucose homeostasis and low‐grade inflammation. Probiotic treatment also led to a reduction in corticosterone release and an increase in the plasma levels of the anti‐inflammatory cytokine IL‐10 in db/db mice (de Cossío et al. 2017).

### 4.3 Metabolites of the Gut Microbiota

#### 4.3.1 SCFAs

**FIGURE 3:**
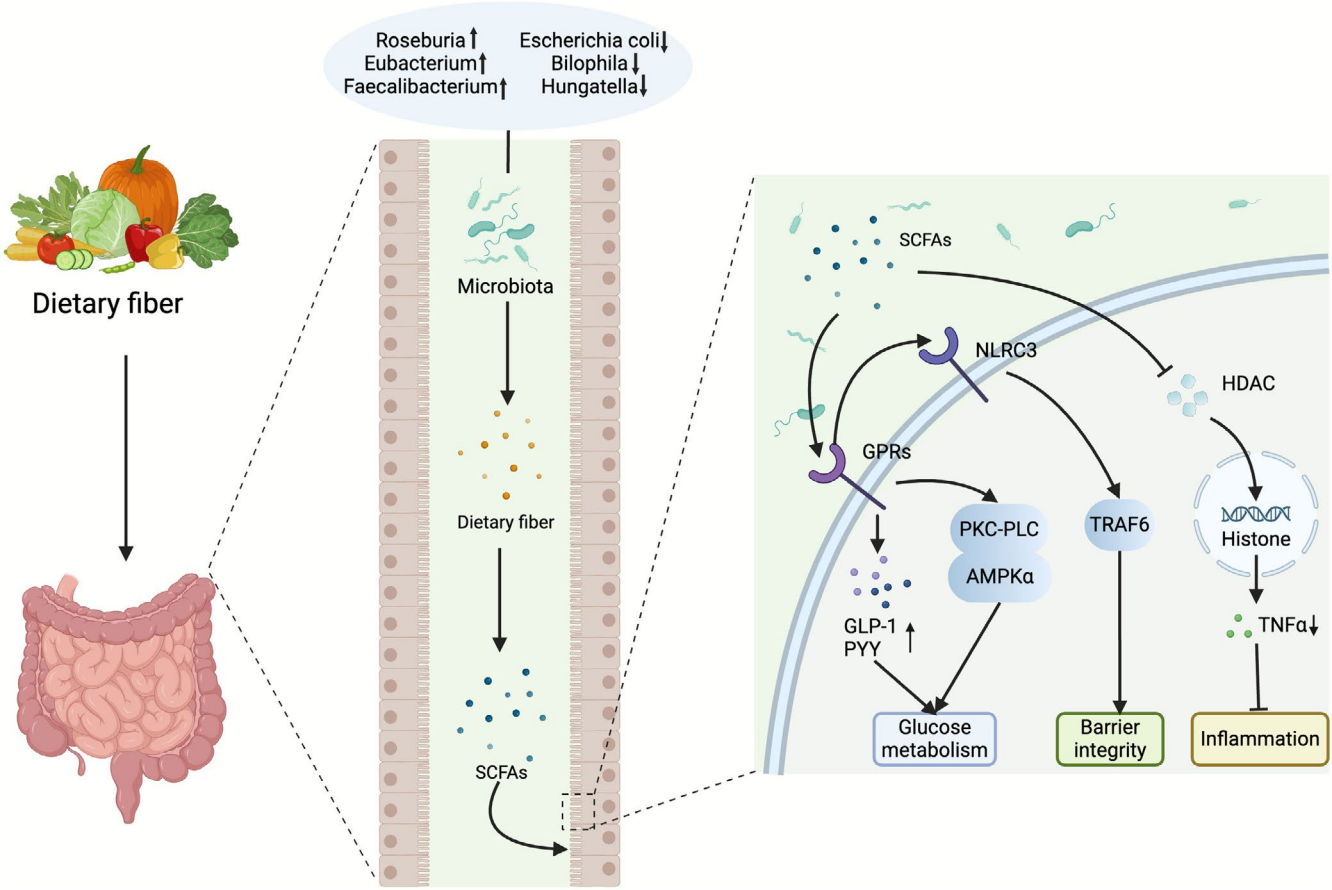
The primary mechanisms through which short‐chain fatty acids (SCFAs) regulate type 2 diabetes mellitus (T2DM). SCFAs enhance glucose metabolism by activating G protein‐coupled receptors (GPRs), promoting the secretion of glucagon‐like peptide‐1 (GLP‐1) and peptide YY (PYY), and inhibiting histone deacetylases (HDACs). Furthermore, SCFAs are closely linked to the protein kinase C‐phospholipase C‐AMP‐activated protein kinase alpha (PKC‐PLC‐AMPKα) signalling pathway and contribute to the maintenance of colonic epithelial barrier integrity in diabetic mice by stimulating the NLRP3 inflammasome in conjunction with GPR43. AMPKα, Adenosine 5′‐monophosphate‐activated protein kinase α; GLP‐1, glucagon‐like peptide‐1; GPRs, G‐protein‐coupled receptors; HDACs, histone deacetylases; NLRC3, NLR family CARD domain‐containing 3; PKC‐PLC, protein kinase C‐phospholipase C; PYY, peptide YY; SCFAs, short‐chain fatty acids; TNFα, tumour necrosis factor α.

SCFAs, primarily acetate, propionate, and butyrate, are the most abundant classes of gut microbial metabolites. These compounds are primarily produced by fermentation of carbohydrates, amino acids, and proteins that remain undigested by the gut microbiota. SCFAs play a crucial role in modulating the interactions between diet, microbiota, and host (Montalvany‐Antonucci et al. 2019; Dai et al. 2010). SCFAs upregulate tight junction proteins, epigenetically regulate immune cells via the HDAC inhibition, modulate intestinal gluconeogenesis, enhance plasma incretin hormone levels, reduce TNFα production, and alter lipid and glucose metabolism (de Groot et al. 2017) (Figure 3). Notably, the faecal concentrations of total organic acids, acetic acid, and propionic acid are significantly lower in patients with T2DM than in controls, and acetic acid and propionic acid concentrations negatively correlate with the duration of T2DM (Sato et al. 2014). Additionally, glucose metabolism is strongly associated with increased levels of SCFA‐producing bacteria, such as *Roseburia*, *Eubacterium*, and *Faecalibacterium* and decreased levels of several pathogenic bacteria, including *Escherichia*, *Bilophila*, and *Hungatella* (Deng et al. 2022). Importantly, butyrate supplementation, without altering food intake, significantly mitigated high‐fat diet‐induced metabolic dysfunctions as evidenced by substantial reductions in weight gain, body fat content, IR, hyperglycaemia, and hyperinsulinaemia (Matheus et al. 2017). A four‐week intervention involving sodium butyrate (SB) treatment in T2DM mice demonstrated that SB significantly improved fasting blood glucose and triglyceride levels. Additionally, it decreased serum levels of LPS, LPS‐binding protein, and pro‐inflammatory cytokines, including IL‐6, TNF‐α, and IL‐1β. Furthermore, SB treatment increased the abundance of butyric acid‐producing bacteria such as *Lachnospiraceae*, *Ruminococcaceae*, *Oscillospira*, and *Ruminococcus* (Wang et al. 2021). Chronic intestinal inflammatory responses are stimulated by nucleotide‐binding oligomerized domain‐like receptors (NLRs), and the overexpression of NLR family CARD domain‐containing 3 (NLRC3) enhances the integrity of the colonic epithelial cell barrier in diabetic mice while upregulating tight junction proteins within the colonic epithelium. Butyrate activates NLRC3 by binding to G protein‐coupled receptor 43 (GPR43) in colonic epithelial cells, thereby improving the integrity of the colonic epithelial barrier and alleviating diabetic symptoms in a TNF receptor‐associated factor 6‐dependent manner (Cheng et al. 2018). *L. plantarum* fermentation enhances the anti‐diabetic properties of *Momordica charantia* juice by promoting the production of acetic acid, propionic acid, butyric acid, and total SCFAs in the colonic contents of diabetic rats (Gao et al. 2019). Probiotics not only maintain microbial diversity but also promote the growth of beneficial bacteria and SCFAs, and their role in reducing IR may be linked to the concentrations of acetic and butyric acids (Ahmadi et al. 2019; Hann et al. 2019). Similarly, after 6 weeks of microbiota infusion from lean body donors, insulin sensitivity in recipients increased along with elevated levels of butyric acid‐producing gut microbiota (Vrieze et al. 2012). The interaction of SCFAs with specific cell signalling pathways may play a key role in the SCFA‐mediated regulation of T2DM pathogenesis. SCFAs are not only used as an essential energy source but also function as signalling molecules that activate GPRs while inhibiting HDAC, thereby improving metabolic homeostasis and energy balance (Gao et al. 2019). Previous studies have shown that activation of GPR43 may have multiple metabolic effects, as the receptor is expressed at high levels in adipocytes, immune cells, gastrointestinal endocrine cells, and pancreatic β‐cells (Lu et al. 2021). SCFA activation of GPRs promotes secretion of GLP‐1 and peptide YY (PYY) by intestinal L‐cells in diabetic rats, which have hypoglycemic functions and are involved in T2DM‐related immunomodulation, thereby improving diabetic symptoms (Yao et al. 2020; Tan et al. 2014; Remely et al. 2014; Sun et al. 2017). Acetic acid‐mediated activation of GPR43 can inhibit insulin bioactivity through protein kinase B (AKT) phosphorylation mediated by the protein kinase C‐phospholipase C‐AMP‐activated protein kinase alpha (PKC‐PLC‐AMPKα) pathway (Lu et al. 2021). The administration of a mixture of *L. rhamnosus LMG S‐28148* and *B. animalis subsp. lactis LMG P‐28149* significantly inhibited the high‐fat diet‐induced decrease in GPR43 and GPR41 expression in insulin‐resistant mice (Alard et al. 2016). Additionally, FMT ameliorates glycolipid disorders by altering the composition of bacteria that produce SCFAs and by activating the GPR43/GLP‐1 P pathway (Han et al. 2021).

SCFAs deficiency is associated with T2DM and negatively correlated with disease duration. An increase in beneficial bacteria in the gut microbiota enhances diabetic symptoms and leads to increased SCFAs levels. Conversely, supplementation with SCFAs improves fasting glucose levels and modulates the structure of the gut microbiota in patients with T2DM. The gut microbiota alleviates diabetic symptoms in T2DM by influencing SCFAs through GPR activation, promotion of GLP‐1 and PYY secretion, and HDAC inhibition. Furthermore, SCFAs are strongly linked to the PKC‐PLC‐AMPKα signalling pathway and enhance the integrity of the colonic epithelial barrier in diabetic mice by binding to GPR43, which stimulates the NLRC3 inflammasome.

#### 4.3.2 BAs

**FIGURE 4:**
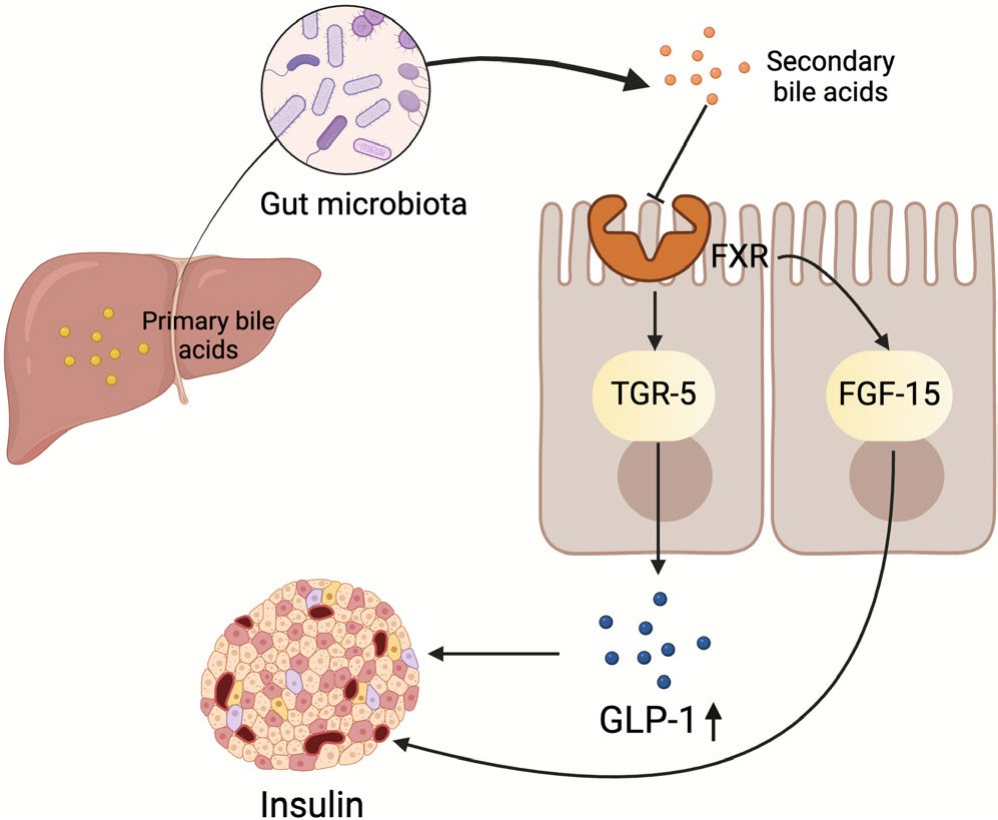
The main mechanisms by which bile acids regulate type 2 diabetes mellitus (T2DM). Primary bile acids are synthesised in the liver and subsequently transformed into secondary bile acids by the gut microbiota. The metabolism of bile acids has been shown to improve T2DM, primarily through the inhibition of FXR and FGF15, as well as the promotion of TGR‐5 and GLP‐1. FGF15, fibroblast growth factor 15; FXR, farnesoid X receptor; GLP‐1, glucagon‐like peptide‐1; TGR‐5, transmembrane G protein‐coupled receptor 5.

BAs metabolism plays a crucial role in regulating host energy, lipid, and glucose metabolism, among other metabolic pathways, primarily through signalling at the farnesoid X receptor (FXR) and transmembrane GPR 5 (TGR‐5) (Wu, Feng, et al. 2021). Disruptions in BA metabolism can impede glucose metabolic pathways, resulting in glucose‐lipid metabolism disorders, and potentially leading to T2DM. Knockdown of the FXR gene in genetically and diet‐induced obese mice reduced hyperglycaemia and hyperinsulinaemia while improving glucose tolerance (Prawitt et al. 2011), whereas TGR‐5 promoted the secretion of GLP‐1, a hormone regulating blood glucose levels (Thomas et al. 2009). Primary BAs, including goose deoxycholic acid, are synthesised in the liver and subsequently converted into secondary BAs, such as deoxycholate and lithocholate, by the gut microbiota, including *Lactobacillus* spp., *Bifidobacterium* spp., *Enterobacteriaceae*, *Mycobacterium* spp., and *Clostridium* spp. (Joyce et al. 2014; Ridlon et al. 2014; Tremaroli and Bäckhed 2012) (Figure 4). Deoxycholic acid is one of the most potent antimicrobial BAs and significantly inhibits the growth of various intestinal bacteria, including *Clostridium perfringens* and *Anaplasma fragilis* (Devlin and Fischbach 2015). Treatment of mice with probiotics such as VSL#3 (including *Lactobacillus*, *Bifidobacterium*, and *Streptococcus*) enhances faecal BA excretion and increases hepatic BA synthesis by downregulating the FXR‐fibroblast growth factor 15(FGF15) signalling axis (Degirolamo et al. 2014). *L. reuteri J1* inhibits weight gain, reduces adiposity, attenuates dyslipidaemia, and improves glucose homeostasis and insulin sensitivity in obese mice. Additionally, *L. reuteri J1* increased the relative abundances of *Lactobacillus* spp., *Akkermann* spp., and *Clostridium* spp., which were strongly correlated with the relative abundances of ursodeoxycholic acid and lithocarbic acid. Ursodeoxycholic acid inhibits FXR expression, whereas lithocarbic acid induces TGR5 expression (Zhang et al. 2022). Consistent with previous studies, WHHPRO (comprising *B. animalis WHH2276*, *L. rhamnosus WHH1155*, *L. fermentum WHH2644*, and WHH39064) demonstrated a hypoglycemic effect and improved glucose metabolism in high‐fat diet fed rats, significantly reversing fasting insulin levels and HOMA‐IR. Furthermore, WHHPRO increased the total unconjugated BA levels in both faeces and the liver and reversed the expression of genes associated with impaired BA metabolic signalling in the ileum and liver. These beneficial effects of WHHPRO are closely linked to the regulation of BA metabolism, FXR‐FGF15 signalling along the hepatointestinal axis, and the composition of the gut microbiome (Cailing et al. 2023). In a 3‐month randomised, double‐blind, placebo‐controlled clinical trial, the combination of ProBio‐X and metformin significantly enhanced the glucose‐lowering effects compared with metformin alone, increased insulin secretion, and improved pancreatic islet function. Both ProBio‐X and metformin elevate BA levels, activating GPRs on the surface of L‐cells, and subsequently promoting GLP‐1 secretion (Chen, Shen, et al. 2023). Therefore, gut microbiota modulators can modulate BA metabolism and improve T2DM symptoms by altering the composition of the gut microbiota, which is associated with the inhibition of FXR‐FGF15 signalling and promotion of TGR5‐GLP‐1 signalling.

#### 4.3.3 BCAAs

**FIGURE 5:**
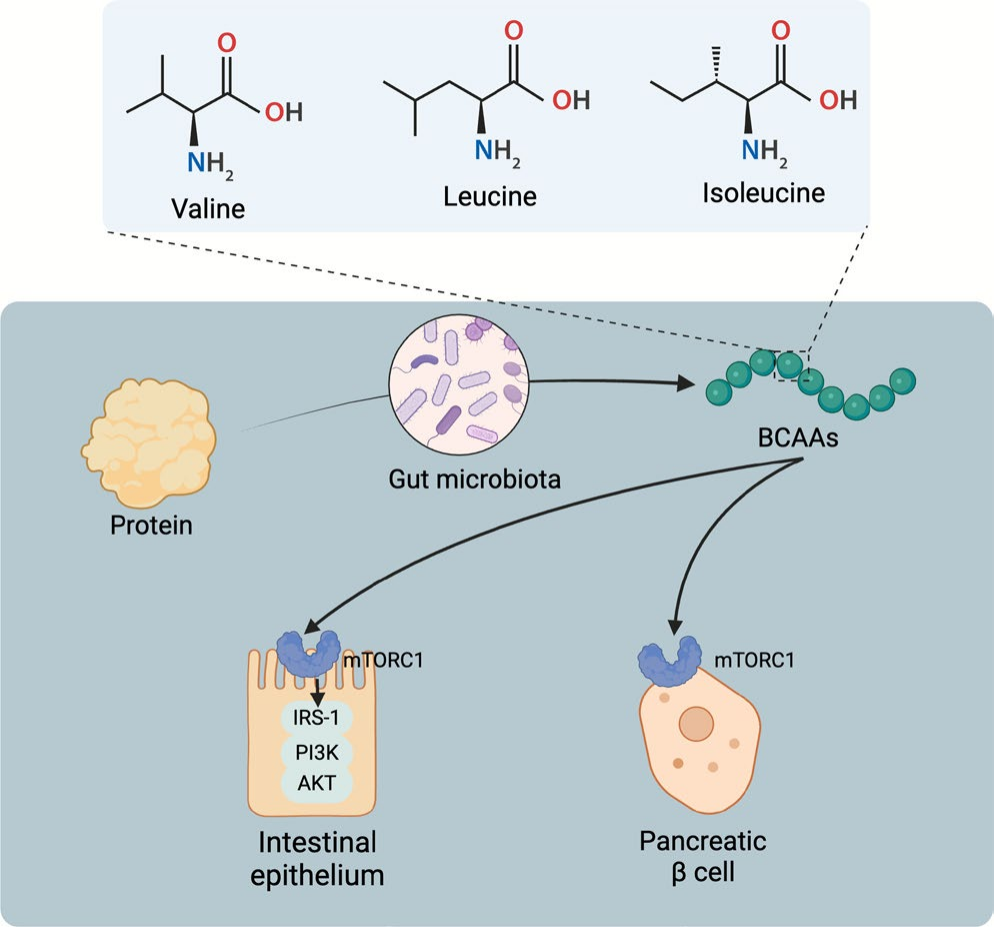
The primary mechanisms through which branched‐chain amino acids (BCAAs) regulate type 2 diabetes mellitus (T2DM). Dysmetabolism of BCAAs leads to increased activation of mTORC1, which can contribute to mitochondrial dysfunction and apoptosis in pancreatic β‐cells. Enhancing the catabolism of BCAAs in the intestine to modulate the intestinal mTORC1/IRS‐1/PI3K/AKT signalling axis has been shown to improve T2DM. AKT, protein kinase B; IRS‐1, insulin receptor substrate‐1; mTORC1, mechanistic target of rapamycin complex 1; PI3K, phosphatidylinositol 3 kinase.

Amino acids are fermented by intestinal bacteria in the distal colon, with protein fermentation yielding higher amounts of BCAAs and potentially toxic substrates, such as ammonia, than carbohydrate fermentation (Rinninella et al. 2019). BCAAs, including leucine, isoleucine, and valine, play critical roles in maintaining the intestinal immune system and activating the intestinal mucosal immune barrier (Zhang et al. 2021). Disruption of BCAA metabolism can lead to the accumulation of toxic metabolic intermediates and increased activation of mammalian target of rapamycin complex 1 (mTORC1), a key regulator of cell growth. This dysregulation can impair insulin signalling (Zhou et al. 2019) and promote mitochondrial dysfunction and apoptosis in pancreatic β‐cells (Hamamah et al. 2024; Lynch and Adams 2014). Enhancing BCAA catabolism in the gut may modulate the intestinal BCAA‐mammalian target of rapamycin complex 1 insulin receptor substrate 1‐phosphatidylinositol 3 kinase‐AKT axis, thereby mitigating the harmful effects of BCAAs on the insulin receptor substrate 1 signalling pathway and improving T2DM (Wang et al. 2024) (Figure 5). Furthermore, disturbances in the gut microbiota have been shown to exacerbate IR and disrupt glucose metabolism by increasing the circulating levels of BCAAs (Pedersen et al. 2016). For instance, Prevotella induces the expression of genes associated with BCAA biosynthesis, while reducing the expression of genes involved in BCAA degradation, contributing to IR in patients (Pedersen et al. 2016). Additionally, the quantity of BCAAs absorbed in the intestine correlates with the abundance of *C. difficile* (Saad et al. 2016). Modifying the composition of the gut microbiota in diabetic mice to reduce BCAA levels can alleviate the symptoms associated with T2DM (Wu, Zuo, et al. 2021). Analysis of faecal specimens from 15 patients with diabetes and 22 healthy controls revealed that the abundance of six bacterial species was significantly elevated in patients with diabetic microangiopathy. Additionally, the levels of valine and isoleucine were elevated, with BCAAs identified as one of the most significant features distinguishing the diabetic microangiopathy group from the control group, following adjustments for estimated glomerular filtration rate and proteinuria (Liu et al. 2023). In conclusion, reducing the BCAA synthesis rate and promoting their degradation by regulating the structure of the gut microbiota may represent an effective therapeutic strategy for T2DM. Furthermore, gut microbiota modulators can alter the species and abundance of specific microorganisms in the intestinal tract, potentially enhancing the management of T2DM. Therefore, the relationship between these agents and BCAAs warrants further investigation.

## 5 Challenges and Future Prospects

The impact of gut microbiota on human diseases is significant and should not be overlooked. Recent studies have investigated how alterations in gut microbes affect human host health and the specific mechanisms involved. T2DM is characterised by complex pathogenesis, and recent studies have demonstrated that gut microbiota modulators modulate gut microorganisms, thereby improving fasting glucose, HbA1c, and IR levels in patients with diabetes. Furthermore, both probiotics and synbiotics have been shown to promote changes in metabolites associated with enhanced metabolic health. Notably, alterations in specific metabolites following synbiotic intervention may confer advantages over probiotic treatment alone (Crovesy et al. 2021). An increasing body of evidence suggests that it is not merely a single strain of gut microbiota that exerts a therapeutic effect, but rather that the entire gut microecology influences health through mutual constraints and balance. These microorganisms coexist and rely on one another in a collective environment. Consequently, future studies should specifically target gut microbiota without disrupting the overall gut microecosystem. FMT is a therapeutic modality involving the transplantation of healthy human gut microbiota into patients. This therapy has the potential to comprehensively modulate the composition of the gut microbiota, thereby providing therapeutic benefits. Studies indicate that FMT is more effective than probiotics in alleviating symptoms of functional bowel disease, and that damage to the intestinal barrier in patients treated with FMT is significantly less than that observed in those receiving probiotic treatment (Ye et al. 2024). Currently, few studies have investigated the number of live bacteria transplanted through FMT treatment, and the precise mechanism by which FMT treatment affects T2DM remains unclear. However, it is important to recognise that changes in one area can influence other aspects of human health. Furthermore, the role of the microbiome may vary depending on the host's condition and genetic factors. Consequently, there is an urgent need to explore microbe‐based diseases from diverse perspectives.

## 6 Conclusion

T2DM is strongly associated with gut microbiota dysbiosis, which is characterised by distinct compositional and abundance variations between T2DM patients and healthy individuals. The pathogenesis of T2DM is closely related to gut microbial disturbances, marked by a reduction in beneficial bacterial species (e.g., *Lactobacillus*, *F. prausnitzii* , and *A. muciniphila* ) and an increase in pathogenic bacteria (e.g., *Bilophila* and *Desulfovibrio*). Probiotics, prebiotics, and synbiotics have demonstrated significant efficacy in improving glycemic control and insulin sensitivity through mechanisms involving the regulation of SCFA‐producing bacteria and the restoration of intestinal barrier integrity. Clinical studies have shown that FMT exhibits hypoglycemic effects comparable to those of metformin. Notably, in patients who continued to exhibit pronounced IR and impaired insulin secretion following metformin treatment, adjunctive FMT enhanced metformin sensitivity. FMT may represent a promising therapeutic option for diabetic patients with multiple comorbidities, suboptimal glycemic control, and polypharmacy. However, several challenges remain. The strain‐specific efficacy of gut microbiota modulators requires further elucidation, and their long‐term safety requires validation in high‐quality clinical trials. Additionally, factors such as donor selection criteria, transplantation methodologies, and baseline gut microbiota variations among patients may influence the therapeutic outcomes of FMT. Collectively, gut microbiota modulation represents an innovative therapeutic approach for T2DM management; however, its clinical implementation requires the integration of personalised treatment strategies and precise regulatory mechanisms.

## Author Contributions

**Jiangyan Wang:** methodology, validation, investigation, data curation, writing – original draft, writing – review and editing, visualization. **Yaofei Wei:** methodology, investigation, writing – original draft. **Dongmian Chen:** investigation, writing – original draft. **Xia Li:** writing – review and editing. **Hao Zhang:** investigation, visualization. **Shuo Feng:** formal analysis. **Shenghua Lu:** investigation. **Juan Yang:** validation, investigation. **Qi Zeng:** investigation. **Xingxiang He:** conceptualization, resources, project administration, funding acquisition. **Lei Wu:** conceptualization, resources, funding acquisition.

## Funding

This work was supported by the Key‐Area Research and Development Program of Guangdong Province, No. 2022B1111070006; the Basic and Applied Basic Research Fund of Guangdong Province, No. 2025A1515011113, 2023A1515012578; the Characteristic Innovation Project of Regular Colleges and Universities in Guangdong Province, No. 2025KTSCX058; the National Natural Science Foundation of China, No. 32202380; the Medical Scientific Research Foundation of Guangdong Province, No. B2022209; the Scientific Research Projects of Guangdong Bureau of Traditional Chinese Medicine, No. 20221232.

## Conflicts of Interest

The authors declare no conflicts of interest.

## Data Availability

The authors have nothing to report.
